# Vegetable and Fruit Intakes Are Associated with hs-CRP Levels in Pre-Pubertal Girls

**DOI:** 10.3390/nu9030224

**Published:** 2017-03-02

**Authors:** Pilar Navarro, Olaya de Dios, Asha Jois, Teresa Gavela-Pérez, Lydia Gorgojo, José M. Martín-Moreno, Leandro Soriano-Guillen, Carmen Garcés

**Affiliations:** 1 Lipid Laboratory, IIS-Fundación Jiménez Díaz, UAM, 28040 Madrid, Spain; pilar.nsanchez@quironsalud.es (P.N.); olaya.dios@quironsalud.es (O.d.D.); ashamaya08@gmail.com (A.J.); TGavela@fjd.es (T.G.-P.); LSoriano@fjd.es (L.S.-G.); 2 Department of Pediatrics, IIS-Fundación Jiménez Díaz, UAM, 28040 Madrid, Spain; 3 Department of Preventive Medicine and Public Health and INCLIVA-Clinical Hospital, University of Valencia, 46010 Valencia, Spain; lydia.gorgojo@gmail.com (L.G.); dr.martinmoreno@gmail.com (J.M.M.-M.)

**Keywords:** hs-CRP levels, pre-pubertal children, fibre intake, vegetable intake, fruit intake, antioxidants

## Abstract

The influence of diet on inflammation in children remains unclear. We aimed to analyze the influence of diet on high-sensitivity C-reactive protein (hs-CRP) levels in a pre-pubertal population free of other influences that may affect hs-CRP levels. We determined hs-CRP levels in 571 six- to eight-year-old children using an hs-CRP ELISA kit. Information on food and nutrient intake was obtained through a food-frequency questionnaire. Overall dietary quality was assessed using the Healthy Eating Index (HEI). We found that girls in the highest tertile of hs-CRP levels had a higher intake of saturated fatty acid, and lower intakes of fiber and vitamin E and a lower HEI score when compared to those in tertiles 1 and 2. We also observed a significant decrease in fruit and vegetable intakes by hs-CRP tertile. Factor analysis showed that a dietary pattern that was loaded most strongly with vegetable, fruit, fiber and vitamin A and E intakes correlated negatively (−0.132, *p* < 0.05) with hs-CRP. No such association was found in boys. In conclusion, our data show that girls with a poorer quality diet show higher hs-CRP levels already at a pre-pubertal age.

## 1. Introduction

Obesity is associated with dysregulation of adipocytokine release, resulting in an increase in the secretion of a number of inflammatory factors that have been associated with an increased cardiovascular risk profile, even in early life [1]. C-reactive protein (CRP) is an acute-phase protein that has been consistently associated with obesity. This is thought to be due to the stimulation of hepatic inflammation by the release of adipocytokines, thereby inducing the production of CRP [2].

It is accepted that pathological processes related to the development of arteriosclerosis, which leads to an increased cardiovascular risk, begin in early life [3,4] and are related to the presence of cardiovascular risk factors at this age. CRP is an inflammatory marker that is considered to activate all stages of atherosclerosis [5].

Diet composition is related to inflammatory status [6]. Several studies suggest an association between diet and CRP levels [7,8]. Oxidative stress has pro-inflammatory effects, and CRP has an important role in this pathway [9], and antioxidant vitamins have been inversely related to CRP concentrations [10,11]. As vegetables and fruits are a major source of antioxidant vitamins, their association with CRP levels has been extensively studied. A number of studies suggest that a high consumption of vegetables and fruits is related to lower levels of CRP [10,12,13]. Studies in children, including the National Health and Nutrition Exmination Survey (NHANES) [14] and the Identification and prevention of Dietary- and lifestyle-induced health EFfects In Children and infantS (IDEFICS) Study in European children [15], have also reported a significant inverse association between a high intake of vegetables and the inflammatory status of the children, particularly CRP levels.

Vegetables and fruit also have high fiber content, and therefore multiple studies have analyzed the potential influence of fiber on plasma CRP levels in adults [16]. Even though epidemiologic studies reported an inverse association between the intake of fiber and levels of hs-CRP [16], a review of the evidence from intervention studies does not support the anti-inflammatory benefits of increasing fiber intake [16].

The relationship between hs-CRP and dietary patterns has also been investigated [17,18]. Dietary patterns poor in vegetable intake and omega-3 fatty acid intake, and high in refined carbohydrates and saturated and trans fatty acids have been associated with high levels of pro-inflammatory molecules in adults [7,17]. The association of plasma CRP levels with a Mediterranean dietary pattern has been largely investigated, and shows a significant association of these patterns with CRP levels [19,20]. However, to our knowledge, no studies analyzing these patterns have been conducted in children. 

In our study we aimed to investigate whether dietary factors are associated with hs-CRP levels in a large, well-defined, population-based sample of pre-pubertal children. We have analyzed the hypothesis that a healthy dietary pattern could be associated with lower hs-CRP levels in children.

## 2. Materials and Methods 

### 2.1. Subjects

Our study included a population-based sample of 571 six- to eight-year-old children (301 girls and 270 boys). Subjects were participants in a cross-sectional study designed to analyze cardiovascular risk factors in Spanish schoolchildren, the Four Provinces Study. Data was gathered cross-sectionally from representative samples of children (age range, six to eight years) in the four cities participating in the study. Children were selected through random cluster-sampling of schools, stratified by sex and socio-economic level (i.e., public versus private schools). Dietary information was gathered on a total of 1,112 children, comprising 557 (50.1%) boys and 555 (49.9%) girls. The overall response rate was 85%, with a similar rate in all four cities [21]. In this study, the population comprises 571 of those children with diet information in whom hs-CRP levels could be measured.

Parents were required to sign a written consent form allowing t heir children to participate. All children reported by their parents to be suffering from metabolic, endocrine, liver or kidney disorders were excluded from the study. Ten children with hs-CRP levels equal or above 10 mg/L were excluded to avoid the influence of acute infection. The study protocol was approved by the Ethics Committee of Clinical Investigation of the Fundación Jiménez Díaz (approval code PI11/00344). The investigation fulfils the principles contained in the Declaration of Helsinki and subsequent reviews, as well as the prevailing Spanish legislation on clinical research in human subjects.

### 2.2. Data Collection

The same team consisting of one physician and several nurses was in charge of blood extractions and physical measurements (weight and height).

### 2.3. Anthropometric Variables

Measurements (weight and height) were taken with fasting children barefoot and wearing light clothing. Height was measured to the last millimetre using a portable stadiometer, and weight was measured to the last 0.1 kg using a standardized electronic digital scale. Body mass index (BMI) (weight in kilograms divided by height in meters squared, kg/m^2^) was calculated from these parameters.

### 2.4. Biochemical Data

Blood samples were obtained early in the morning after a 12 h fasting period by venipuncture. Samples were kept on ice and sent to the laboratory for analysis. Once centrifuged, fractions were separated and frozen at −70 °C for future analyses. CRP levels were measured using a high sensitivity C-Reactive Protein ELISA kit (SK00080-02, Aviscera Bioscience, Inc., Santa Clara, CA, USA). The sensitivity of the assay was 0.15 mg/L. The intra-assay and inter-assay precision were between 4%–6% and 8%–12%, respectively.

### 2.5. Food and Nutritional Data

The relationship between hs-CRP levels and diet was evaluated by analyzing the association between hs-CRP with nutrients, food items, HEI and whole dietary patterns determined by factor analysis. Information on food and nutrient intake was obtained through a food-frequency questionnaire (FFQ) initially developed for use on adults and previously validated in Spain by Martín-Moreno and cols [22]. For the purpose of this study, the questionnaire was adapted to a primary school population by amending and downscaling the list of foods and portions consumed on the basis of a systematic review of child-population food surveys in Spain [23].

The final version of the questionnaire included a total of 77 food codes grouped into 11 categories by nutrient composition. For each food, the standard serving size was defined (e.g., one cup of milk equivalent to 170 cc.; a dish of lentils, equivalent to 60 g dry weight) and the mean frequency of consumption of such servings over the previous year was ascertained. The questionnaire provided the option of answering, in an open-ended way, in terms of the frequency per day, week, month or year. Using Spanish food-composition tables, a food frequency conversion program was designed, which furnished a database with the annual food consumption and daily nutrient intake frequencies for each individual surveyed.

Overall dietary quality was assessed using the HEI [24]. This index consists of 10 items: five items measure food groups (cereals, vegetables, fruit, dairy products and meat); four measure nutrients (total lipid, saturated fat, cholesterol and sodium); and one item analyzes dietary variety. For each HEI item, a score is obtained between 0 and 10 on a proportional basis. The total score, which can range anywhere from 0 to 100, is obtained by adding up the individual scores for the 10 items; the higher the score the better diet quality.

### 2.6. Statistical Analysis

Statistical analysis was performed using the SPSS software package, version 21.0 (SPSS, Inc., Chicago, IL, USA). As the sensitivity of the assay was 0.15 mg/L, observations of hs-CRP within the interval of concentrations from 0 to 0.15 mg/L (33% of the children) were set to 0.15 mg/L. Children with hs-CRP levels equal or above 10 mg/L were excluded of the analysis. As a sex-dependent association between diet and hs-CRP has been previously described [15], sex-stratified analyses were performed. Any dietary variables that were not normally distributed were log transformed to normality prior to analyses. We used a *t*-test or Mann-Whitney test to compare nutrient intake by sex. ANOVA or Kruskal-Wallis test and the corresponding Post Hoc test were used to compare the means of nutrient and food intake depending on hs-CRP levels tertile. To identify major dietary patterns experimental factor analysis was conducted. The identified factors were rotated using a Varimax rotation to ensure that standardized, non-correlated dietary patterns were identified. The Kaiser–Meyer–Olkin (KMO) test was used to evaluate data adequacy for factor analysis. Eigen values higher than one and the Screen plot determined whether the factor should be retained. Regression factor scores were used for partial correlation analyses investigating the association between dietary patterns and hs-CRP.

## 3. Results

The sample group was made up of 571 children (301 girls and 270 boys), with an average age of 6.7 years. Hs-CRP levels were higher in girls than in boys (Table 1). The energy and percentage of energy from saturated fat intake were slightly lower in girls than in boys, and the percentage of complex carbohydrate intake was slightly higher (Table 1).

### 3.1. Nutrient Intake and hs-CRP Levels

We analyzed the nutrient intake by tertile of hs-CRP levels after adjusting for BMI (Table 2). In girls, those in the highest hs-CRP tertile (hs-CRP ≥ 0.62 mg/L) had a higher intake of saturated fatty acid, and lower intakes of fiber and vitamin A and E when compared to those in tertiles 1 (hs-CRP ≤ 0.15 mg/L) and 2 (hs-CRP levels between 0.16–0.61 mg/L). Girls in the highest hs-CRP tertile had a significantly lower intake of fiber (17.9 ± 6.3 g/day) compared to those in the lowest tertile (20.8 ± 7.3 g/day). Vitamin E intake in the highest hs-CRP tertile was significantly lower than the intake in tertiles 1 and 2. The HEI score of the highest tertile of hs-CRP levels in girls (62.8 ± 10.3) was also significantly lower than the HEI score in the lowest tertile (66.1 ± 8.9) (Table 2).

These differences were not apparent in boys.

### 3.2. Food Consumption and hs-CRP Levels

**Figure 1:**
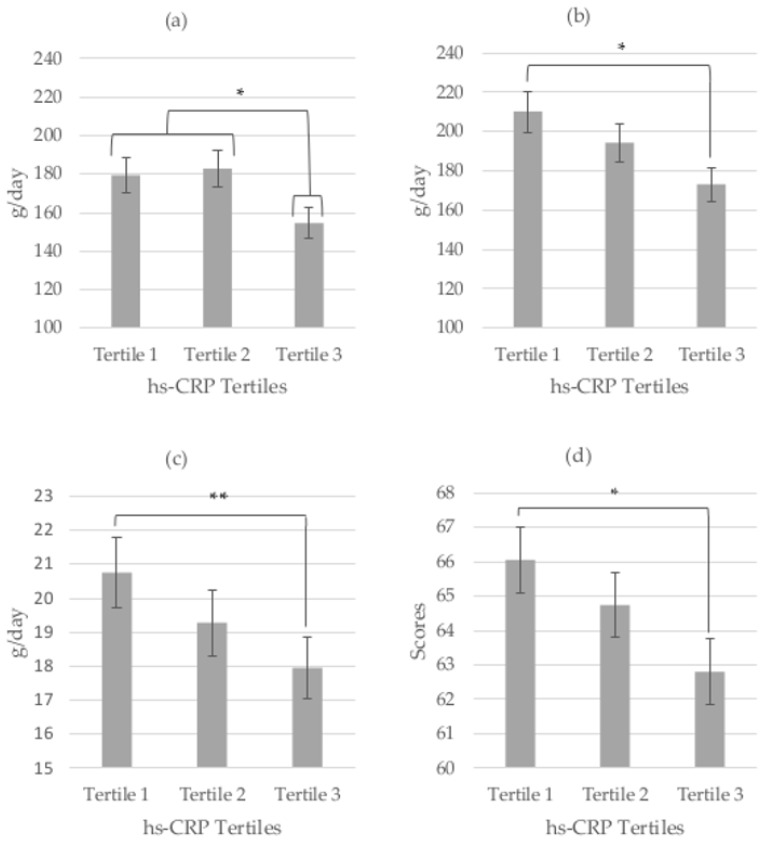
(**a**) Vegetable, (**b**) fruit and (**c**) fiber intakes and (**d**) HEI score by hsCRP tertiles in pre-pubertal girls after adjusting for BMI. (ANOVA and Tukey or Games–Howell Post Hoc test, *p*-value: * *p* < 0.05, ** *p* < 0.01).

In accordance with the decrease in fiber by hs-CRP tertile described above, we observed a decrease in fruit and vegetable intakes by hs-CRP tertiles in girls (Figure 1). Girls in the highest hs-CRP tertile had a significantly lower intake of vegetables compared to those in tertiles 1 or 2 (154.6 g/day in the tertile 3 versus 181.0 g/day in the tertiles 1–2, *p* < 0.05). Girls in the highest hs-CRP tertile had a significantly lower intake of fruit (173.2 g/day) compared to those in tertile 2 (194 g/day) or tertile 1 (210 g/day) (*p* < 0.05). Again, no differences were found in boys.

When analyzing specific foods, we observed that marmalade intake was significantly related to hs-CRP levels, with the highest intake associated with the lowest hs-CRP tertile when compared to the higher tertiles (1.6 g/day in the highest tertile versus 3.5 g/day in the lowest; *p* < 0.05).

### 3.3. Dietary Patterns and hs-CRP Levels

To identify dietary patterns that could be related to hs-CRP levels, principle component factor analysis was performed. Examination of the subsequent Kaiser–Meyer–Olkin (KMO) measure of sampling adequacy showed that the sample was factorable (KMO = 0.662). By examining the Screen plot and Eigen values, four factors were selected in our population. Two main factors explained most of the variance in food intake. Factor 1 explained 26% of the variance in food intake. It loaded most strongly on fiber, fruit, fruit and vegetable, and vitamin A and E intakes (Table 3). This factor had a recognizably ‘high vegetable intake’ pattern. The second factor explained another 25% of the variance in food intake. It loaded most strongly on fat intake, particularly monounsaturated and polyunsaturated intakes. We termed this diet the ‘high fat’ diet (Table 3). The high vegetable intake pattern correlated negatively (−0.132, *p* < 0.05) with hs-CRP in girls after adjusting by BMI. No associations were found between hs-CRP levels and the high fat diet. No correlations for either of the patterns were found in boys.

## 4. Discussion

We measured hs-CRP levels in a large, population-based sample of pre-pubertal children in Spain, and identified a number of dietary factors associated with hs-CRP levels in girls. Our results show that a high intake of saturated fat and a low intake of fiber and vitamins are related to the highest hs-CRP levels in girls. Furthermore, our findings emphasize that a low HEI score is associated with the highest hs-CRP levels. We also identified a “high vegetable intake dietary pattern” that is associated with lower hs-CRP levels. Our study adds to the literature that has investigated this association in children, finding an association of hs-CRP levels with a characteristic high fiber, high vegetable intake pattern in a well-defined cohort of pre-pubertal girls. The association of fat intake with hs-CRP levels has been extensively studied in adults [7,8,25], and there have been several studies in children [26,27,28]. Cross-sectional studies have found a positive association between trans-fat or saturated fat intake and CRP levels [7,8], but this has not yet been confirmed in interventional studies. Thus, the results regarding the association between specific types of fat intake and hs-CRP levels in adults remain inconclusive [8]. Similar to our findings, a positive association of saturated fat intake with CRP has been described in six- to 14-year-old Swiss children [26], although the association has not been found in older girls [27].

The association between a higher intake of fruit and vegetables and lower hs-CRP levels appears to be consistent in both adults [10,11] and children [14,15]. However, the association seems to depend on age and sex [15] and weight category status [10]. Sex-related differences in the association of hs-CRP levels with diet characteristics were present in our study, but no differences by weight category were found. Our findings show that a high intake of fruit and vegetables is associated with lower hs-CRP levels in girls only. In some studies in adults, the association has been described only in men but not in women [10]. The IDEFICS Study in European children described differences in the association between hs-CRP and vegetable intake by both age group and sex [15], reporting an association with raw vegetables in boys and older girls and with cooked vegetables in younger girls.

The reason behind the different association depending on sex in our children is unclear. Children in our study showed no significant difference in mean BMI or in the intake of fiber, vitamins or vegetables, and are all pre-pubertal and therefore lacking potential hormonal influences that could affect this association. It is possible that the differences could be attributed to the influence of hormones such as leptin on energy homeostasis. Leptin levels are significantly higher in girls than in boys [29] and have been related to hs-CRP levels in our pre-pubertal population [30].

The high fiber content and high antioxidant levels related to vegetables and fruit may contribute to explaining the association between vegetable and fruit intake and hs-CRP levels. In our population we confirmed a significantly high fiber intake in girls with lower hs-CRP levels. The association of fiber with CRP levels has been reported in some studies [31,32], although a review of the literature has shown that even though the association is evident in epidemiologic studies, intervention studies failed to find any significant association [16]. To our knowledge, how fiber has the potential to modulate hs-CRP levels is unknown. It may be related to its effects on slowing glucose absorption and in modulating the production of inflammatory cytokines by gut microflora [33].

Furthermore, an inverse association of hs-CRP with vitamin A and vitamin E has been reported in our study, supporting the fact that antioxidant nutrients may be responsible for lowering hs-CRP levels. In support of this theory, some studies in adults have reported inverse associations between hs-CRP and other antioxidants, such as vitamin C, vitamin A and β-carotene [10,11,13], although the association has not been found in children [27].

Interestingly, specific foods such as marmalade were significantly associated with hs-CRP levels in our girls. Unfortunately, in our study we lack information regarding the type of marmalade children are consuming. However, similar to our results, a significant inverse association between honey/jam intake and CRP levels was described in the IDEFICS Study in European children [15].

In our study, the overall dietary quality was measured using the HEI [24]. We found an inverse, significant association between hs-CRP with HEI in girls. The HEI score was associated with a higher vegetable intake and a lower fat intake. An inverse association of CRP levels with HEI has also been reported in other studies in adults [34,35] and children [36,37].

Consistent with the results discussed previously, we found that a high vegetable intake pattern had a significant negative association with hs-CRP levels in girls. Our pattern was defined by a high fiber intake, a high intake of fruit and vegetables, and a high intake of vitamins A and E. In studies in adults, similar patterns have been negatively associated with hs-CRP levels [17,32]; however, to our knowledge; the association of dietary patterns with hs-CRP levels has not previously been studied in children.

The main limitation of our study was the method of collecting dietary information. FFQs introduce the potential for recall bias, which is of particular importance in children. We countered this issue by obtaining dietary information from the primary carer of each child, trying to minimize recall bias. Particularly debatable is the definition of the terms “simple and complex carbohydrates”. In addition to fiber, complex carbohydrates in our study included several forms of carbohydrates, including starches. This may contribute to the lack of a significant association between hs-CRP levels and complex carbohydrates, despite our finding of an association between hs-CRP and fiber.

In conclusion, we showed that specific dietary factors—namely saturated fat, fruit and vegetable and fiber intake, and vitamins A and E—were associated with variations in hs-CRP levels in girls, independent of BMI. We identified a high vegetable intake pattern that negatively correlated with hs-CRP. These findings suggest that diet seems to be related to hs-CRP levels already at the pre-pubertal age, emphasizing the need for dietary guidelines in preventing high hs-CRP levels from childhood.

## Figures and Tables

**Table 1:** Anthropometrics measures, hs-CRP levels (mean ± SD) and daily nutrient intake in children by sex.

Variable	Girls (*n *= 301)	Boys (*n *= 270)
Age (years)	6.7 ± 0.7	6.7 ± 0.6
Weight (kg)	27.4 ± 5.9	27.5 ± 5.6
Height (m)	1.26 ± 0.07	1.27 ± 0.06
BMI	17.2 ± 2.7	17.2 ± 2.6
*z*-Score BMI	0.01 ± 0.96	0.05 ± 0.98
hs-CRP (mg/L)	1.05 ± 1.74	0.87 ± 1.55 *
Energy (Kcal)	2090 ± 497	2186 ± 518 *
Protein energy (%)	17.2 ± 2.4	17.3 ± 2.5
Total fat energy (%)	45.7 ± 4.5	46.4 ± 4.6
Saturated-fat energy (%)	16.4 ± 2.7	17.2 ± 2.8 **
Monounsaturated-fat energy (%)	18.4 ± 2.7	18.3 ± 2.6
Polyunsaturated-fat energy (%)	8.3 ± 1.8	8.2 ± 1.9
Total carbohydrate energy (%)	38.4 ± 5.8	37.8 ± 6.0
Complex-carbohydrate energy (%)	18.0 ± 4.5	17.3 ± 4.1 *
Simple-carbohydrate energy (%)	21.1 ± 5.6	21.3 ± 6.1
Fiber (g)	19.6 ± 7.2	19.9 ± 7.6

*t*-test or Mann–Whitney Test when required, * *p* < 0.05, ** *p* < 0.01. Simple carbohydrates: Sugars in milk, fruit and sugar cane. Complex carbohydrates: Fiber and starches in whole grains, legumes, nuts, seeds and vegetables.

**Table 2:** Daily energy, nutrient intakes and HEI score (mean ± SD) by hs-CRP tertile in pre-pubertal children by sex, adjusted for BMI.

Nutritional Variable	Girls				Boys			
	Tertile 1 (≤0.15 mg/L)	Tertile 2 (0.16–0.61 mg/L)	Tertile 3 (≥0.62 mg/L)	*p* Value	Tertile 1 (≤0.15 mg/L)	Tertile 2 (0.16–0.61 mg/L)	Tertile 3 (≥0.62 mg/L)	*p* Value
Energy (kcal)	2083 ± 446	2104 ± 531	2021 ± 492	NS	2162 ± 542	2173 ± 510	2223 ± 539	NS
Protein (%)	16.9 ± 2.5	17.3 ± 2.7	17.3 ± 2.5	NS	16.9 ± 2.7	17.1 ± 2.3	17.5 ± 2.4	NS
Total fat (%)	45.3 ± 4.8	46.3 ± 4.2	45.7 ± 4.3	NS	46.0 ± 4.3	46.7 ± 4.5	45.6 ± 4.3	NS
Saturated-fat (%)	15.7 ± 2.7	16.6 ± 2.3	17.1 ± 3.0	1–3 **	17.3 ± 2.8	17.0 ± 2.7	16.9 ± 2.7	NS
Monounsaturated-fat (%)	17.9 ± 2.5	18.5 ± 2.8	18.7 ± 2.9	NS	18.1 ± 2,4	18.5 ± 2.5	18.0 ± 2.4	NS
Polyunsaturated-fat (%)	8.5 ± 1.9	8.5 ± 1.8	8.1 ± 1.8	NS	8.1 ± 1.8	8.5 ± 2.1	7.9 ± 1.8	NS
Total carbohydrate (%)	39.3 ± 6.1	37.7 ± 5.3	38.4 ± 5.8	NS	38.4 ± 5.9	37.5 ± 5.6	38.6 ± 5.7	NS
Simple-carbohydrate (%)	21.6 ± 5.1	20.8 ± 5.9	21.3 ± 6.5	NS	21.9 ± 6.1	21.1 ± 5.4	21.7 ± 5.8	NS
Complex-carbohydrate (%)	18.1 ± 4.5	17.8 ± 5.1	17.7 ± 3.9	NS	17.4 ± 3.9	17.5 ± 4.6	17.5 ± 4.1	NS
Fiber (g)	20.8 ± 7.3	19.3 ± 7.7	17.9 ± 6.3	1–3 **	19.9 ± 7.4	19.5 ± 6.2	20.1 ± 6.5	NS
Vitamin A (μg)	646 ± 169	659 ± 170	616 ± 206	NS	706 ± 207	693 ± 191	700 ± 212	NS
Vitamin E (mg)	10.8 ± 3.5	10.9 ± 4.3	9.8 ± 4.2	1–3 *	10.8 ± 4.7	10.9 ± 3.7	10.6 ± 3.9	NS
				2–3 *				
HEI score	66.1 ± 8.9	64.7 ± 8.5	62.8 ± 10.3	1–3 *	63.4 ± 8.6	64.3 ± 7.6	65.4 ± 7.9	NS

%: percentage of total caloric intake supplied by the nutrient; HEI: Healthy Eating Index; NS: not significant; *p* value: * *p* < 0.05, ** *p* < 0.01, ANOVA adjusting for BMI with the Tukey or Games–Howell post hoc test.

**Table 3:** Factor-loading matrix of two dietary patterns (high vegetable intake pattern and high fat intake dietary pattern) identified in our population by factor analysis.

Nutritional Variable	Dietary Patterns as Detected by Factor Analysis	
	Factor 1: High Vegetable Intake Diet	Factor 2: High Fat Intake Diet
Energy	0.786	−0.160
Protein energy (%)	−0.156	0.163
Total fat energy (%)	−0.127	0.921
Saturated fat energy (%)	−0.446	0.217
Monounsaturated fat energy (%)	−0.002	0.782
Polyunsaturated fat energy (%)	−0.005	0.712
Total carbohydrate energy (%)	0.195	−0.853
Complex carbohydrate energy (%)	0.009	−0.384
Simple carbohydrate energy (%)	0.129	−0.538
Fiber	0.814	−0.307
Vitamin A	0.521	0.071
Vitamin E	0.779	−0.002
Fruit	0.711	−0.239
Fruit and vegetables	0.785	−0.267

Coefficients with a significant contribution to the model (values ≥ 0.30) have been underscored.
